# Association of heart rate variability and inflammatory response in patients with cardiovascular diseases: current strengths and limitations

**DOI:** 10.3389/fphys.2013.00174

**Published:** 2013-07-10

**Authors:** Vasilios Papaioannou, Ioannis Pneumatikos, Nikos Maglaveras

**Affiliations:** ^1^Intensive Care Unit, Alexandroupolis General Hospital, Democritus University of ThraceAlexandroupolis, Greece; ^2^Laboratory of Medical Informatics, School of Medicine, Aristotle University of ThessalonikiThessaloniki, Greece

**Keywords:** heart rate variability, inflammation, autonomic nervous system, coronary artery disease, cardiovascular disease, mortality

## Abstract

Many experimental and clinical studies have confirmed a continuous cross-talk between both sympathetic and parasympathetic branches of autonomic nervous system and inflammatory response, in different clinical scenarios. In cardiovascular diseases, inflammation has been proven to play a pivotal role in disease progression, pathogenesis and resolution. A few clinical studies have assessed the possible inter-relation between neuro-autonomic output, estimated with heart rate variability analysis, which is the variability of R-R in the electrocardiogram, and different inflammatory biomarkers, in patients suffering from stable or unstable coronary artery disease (CAD) and heart failure. Moreover, different indices derived from heart rate signals' processing, have been proven to correlate strongly with severity of heart disease and predict final outcome. In this review article we will summarize major findings from different investigators, evaluating neuro-immunological interactions through heart rate variability analysis, in different groups of cardiovascular patients. We suggest that markers originating from variability analysis of heart rate signals seem to be related to inflammatory biomarkers. However, a lot of open questions remain to be addressed, regarding the existence of a true association between heart rate variability and autonomic nervous system output or its adoption for risk stratification and therapeutic monitoring at the bedside. Finally, potential therapeutic implications will be discussed, leading to autonomic balance restoration in relation with inflammatory control.

## Introduction

Systemic inflammation is a normal response to altered homeostasis and has an important role in several pathophysiological processes, such as infection or trauma. It is characterized by the endocrine release of different cytokines, such as tumor necrosis factor α (TNF-α) and interleukin-1 (IL-1), IL-4, IL-6, IL-10, and many others, normally confined to paracrine regulation of a local inflammatory response (Koj, 1997; Sporn, 1997). Apart from their involvement in local and systemic inflammation, cytokines may induce activation of brain-derived neuroendocrine immunomodulatory responses. Neuro-endocrine pathways, such as hypothalamo-pituitary-adrenal (HPA) axis and both the sympathetic and parasympathetic divisions of the autonomic nervous system (ANS) are powerful modulators of inflammation, typically through an anti-inflammatory balancing mechanism (Reichilin, 1993; Webster et al., 2002).

Recently, it has been demonstrated that subclinical inflammation and the con-centration of inflammatory markers, such as cytokines, correlate strongly to cardio-vascular mortality and morbidity in both healthy subjects and in those with known coronary artery disease (CAD) (Phillips et al., 1992; Ridker et al., 1997). Furthermore, vascular inflammation plays a critical role in the initiation, evolution, and rupture of atherosclerotic plaque (Ross, 1993).

In the healthy state there is some degree of stochastic variability in physiologic variables, such as heart rate (heart rate variability). This variability is a measure of complexity that accompanies healthy systems and has been suggested to be responsible for their greater adaptability and functionality related to pathologic systems (Buchman, 2002). Studying physiological signals of patients can easily identify ”hidden” information concerning inherent dynamics and overall variability within a time series. Recognition that physiologic time series contain such information defies traditional mechanistic approaches based on conventional biostatistical methodologies and has fueled growing interest in applying techniques from statistical physics for the study of living organisms (Seely and Christou, 2000). Through those techniques different “physiomarkers” can be estimated that fulfill the requirements of contemporary medicine for better and more accurate early warning signs, since they are based on high-frequency measurements and are much easier to measure at the bedside (Seely and Christou, 2000). In this respect, a number of international databases and different processing methods of heart rate signals have been developed with free access from different investigators, such as the Web Site Physionet (www.physionet.org).

On the contrary, it has been repeatedly demonstrated that various “biomarkers” such as cytokines, exhibit marked interdependence, pleiotropy (multiple effects) and redundancy (multiple cytokines with the same effect) (Friedland et al., 1996). At the same time, their plasma concentrations fluctuate from day to day and correlate poorly with classic physiologic variables in different groups of patients (Friedland et al., 1996; Seely and Christou, 2000; Buchman, 2002). Furthermore, biomarkers are difficult to obtain routinely at the bedside. In addition, the financial cost of various immunoassay techniques for their detection in blood samples tends to become an inhibiting factor for their extensive use, as diagnostic or even prognostic tools, in many Medical Centers.

## Neuro-immunological cross-talk and heart rate variability analysis

The pathophysiological link between the communication between the Central Nervous System (CNS) and the immune-regulated inflammation is the capability of the brain to monitor and to affect at the same time the immune status. The first mechanism relies upon activation of vagus nerve afferent fibers that signal the brain that inflammation is occurring. Different kind of mediators such as cytokines can activate visceral vagus afferent fibers which terminate within the dorsal vagal complex (DVC) of the medulla oblongata. The DVC consists of the nucleus tractus solitarius (NTS), the dorsal motor nucleus of the vagus (DMN) and the area postrema (AP) (Berthhoud and Neuhuber, 2000). Ascending projections from the NTS reach hypothalamic paraventricular nucleus (PVN), which is associated with the synthesis and release of corticotropin releasing hormone (CRH). This factor induces the production of adrenocorticotropin hormone (ACTH) from the anterior pituitary, which is the main inducer of the synthesis of immuno-suppressive glucocorticoids from the adrenal cortex. Projections from NTS are connected to the DMN and to rostral ventrolateral medulla (RVLM). This region increases firing of the noradrenergic preganglionic neurons in the spinal cord (Tracey, 2007).

The brain can affect the immunological status through the activation of the HPA axis and increased outflow of sympathetic (SNS) and parasympathetic nervous system. The SNS activation during the early stages of stress induces local inflammatory response through α_2_–subtype adrenoreceptor stimulation by norepinephrine (NE), whereas stimulation of β_2_-subtype adrenoreceptor-cAMP-protein kinase A pathway is associated with an inhibition of pro-inflammatory cytokines' production (van der Poll et al., 1996; Elenkov et al., 2000; Zhou et al., 2001). It seems that SNS activation protects the organism from the detrimental effects of pro-inflammatory cytokines, while it can increase local inflammatory response (Chrousos, 1995). In addition to the SNS, a link between the parasympathetic part of the ANS and immune-regulatory processes has been suggested (Tracey, 2002). It has been demonstrated that acetyl-choline decreases TNF-α production by endotoxin-stimulated human macrophage cultures, through α 7-subunit of the nicotinic acetylcholine receptor (Wang et al., 2003; de Jonge et al., 2005). The vagus nerve cholinergic signaling interacts with the above receptor on immune cells in the spleen and inhibits TNF-α production and release into the circulation (Huston et al., 2006). Acetylcholine is also effective in suppressing other pro-inflammatory cytokines such as IL-1β, IL-6, and high mobility group box 1 (HMGB1) protein (Wang et al., 2004). This ”cholinergic anti-inflammatory pathway” is responsible for a ”hard-wired” connection between the nervous and immune systems and is considered, as the primary component of the “immuno-reflex.” A more complete understanding of these reflexes can yield insight into both pathophysiological pathways and therapeutic strategies in many pathological processes, including infections, sepsis and cardiovascular diseases (Tracey, 2002, 2007).

In conclusion, there is strong evidence that CNS controls body's systemic response to inflammation. Recently, different clinical studies investigating a possible association between ANS outflow and various inflammatory indices in patients with heart diseases have appeared in the literature (Aronson et al., 2001; Malave et al., 2003; Janszky et al., 2004; Shehab et al., 2004; Hamaad et al., 2005; Lanza et al., 2006; Madsen et al., 2007; Nolan et al., 2007; Psychari et al., 2007; von Känel et al., 2011). The aim of these studies was to measure ANS activity through a set of different “physio-markers” and correlate them with various biomarkers that can indirectly assess inflammatory response in different clinical scenarios, such as CAD (Janszky et al., 2004; Hamaad et al., 2005; Lanza et al., 2006; Madsen et al., 2007; Nolan et al., 2007; Psychari et al., 2007; von Känel et al., 2011) and heart failure (Aronson et al., 2001; Malave et al., 2003; Shehab et al., 2004). Moreover, the prognostic value of such measurements was tested in different groups of patients with cardiovascular diseases, in terms of mortality and risk of rehospitalization.

The best “physiomarkers” are obtained from analysis of heart rate variability (HRV); that is, the variability of R-R series in the electrocardiogram (ECG), and its frequency components (Lombardi et al., 1987; Task Force, 1996). Beat-to-beat fluctuations reflect the dynamic response of the cardiovascular control systems to a host of naturally occurring physiological perturbations. A variety of animal and human research has established two clear frequency bands in heart rate signals. These bands include high frequency oscillations, between 0.15 and 0.4 Hz that are associated with respiration, and bands with a lower frequency range, below 0.15 Hz (Task Force, 1996). Akselroad et al. (1981) introduced power spectrum analysis of heart rate fluctuations in order to quantify beat-to-beat cardiovascular control. Power spectrum density (PSD) analysis provides the basic information of how power (variance) distributes as a function of frequency (Akselroad et al., 1981; Malik and Camm, 1993). In 1996, the Task Force of the European Society of Cardiology and the Northern American Society of Pacing and Electrophysiology published guidelines regarding standardization of nomenclature, specification of methods of measurement, definition of physiological and pathophysiological correlates, description of clinical applications and identification of different areas for future research.

The association of higher risk of post-infarction mortality with reduced HRV was first shown by Wolf et al. (1978). The clinical importance of HRV became appreciated in the late 1980s, when it was demonstrated that low HRV was a strong and independent predictor of mortality after an acute myocardial infarction (MI) (Kleiger et al., 1987; Lombardi et al., 1987).

## Measuremeny of heart rate variability

The RR variations may be evaluated by a number of methods:

### Time domain methods

Time domain methods determine heart rate or RR intervals in continuous ECG records. Each QRS complex is detected and the normal-to-normal (NN) intervals (all intervals between adjacent QRS complexes) are calculated. Other time domain variables include the mean NN interval, the mean heart rate or the difference between the longest and the shortest NN interval, as well. The simplest of these metrics is the standard deviation of the NN intervals (SDNN), which is the square root of the variance. However, it should be emphasized that SDNN becomes less accurate with shorter monitoring periods. The most commonly used time domain methods are the square root of the mean squared differences of successive NN intervals (RMSSD), the number of interval differences of successive NN intervals greater than 50 ms (NN50) and the proportion derived from dividing NN50 by the total NN intervals (pNN50) (Akselroad et al., 1981; Task Force, 1996; Table 1).

**Table 1:** **HRV metrics in time domain**.

SDNN	Standard deviation of all N-N intervals
SDNN index	Average of the standard deviations of N-N intervals for each 5-min period
SDANN	Standard deviation of the average N-N intervals for each 5-min period over 24 h
NN50	Number of N-N intervals differing by >50 ms from the preceding interval
pNN50	Percentage of adjacent cycles that are >50 ms apart
RMSSD	Root mean square of successive differences in ms

### Frequency domain methods

Spectral analysis of heart rate partitions HRV into its frequency components. Most commonly used methods are Fast Fourier Transformation (FFT) and auto-regressive modeling. FFT displays in a plot the relative contribution (amplitude) of each frequency. This plot includes at least three peaks.Fast periodicities in the range 0.15–0.4 Hz [high frequency (HF)] are largely due to the influence of the respiratory phase on vagal tone. Low-frequency periodicities (LF), in the region of 0.04–0.15 Hz, are produced by baroreflex feedback loops, affected by both sympathetic and para-sympathetic modulation of the heart, whereas very low frequency periodicities (VLF), in the frequency range between 0.003 and 0.04 Hz and ultra low frequencies (ULF, <0.003 Hz) have been variously ascribed to modulation by chemoreception, thermo-regulation and the influence of vasomotor activity. The area under the power spectral curve (power) in a particular frequency band is considered to be a measure of HRV at that frequency, whereas the LF/HF ratio has been suggested as an indirect index of sympathovagal balance (Task Force, 1996). According to the report of the Task Force, the analyzed ECG signals must satisfy several technical requirements in order to obtain reliable information. The optimal sampling frequency range should be between 250 to 500 Hz. Ectopic beats, arrhythmic events, missing data and noise effects should be properly filtered and omitted. Frequency domain methods must be preferred in cases of short term investigations. The recordings should last for at least 10 times the wavelength of the lower frequency bound, thus recordings of ~1 min can assess the HF component of HRV while 2 min are needed for the LF component. In conclusion, 5-min recordings are preferred, unless the aim of the study dictates a different design (Akselroad et al., 1981; Lombardi et al., 1987; Task Force, 1996; Table 2).

**Table 2:** **HRV metrics in frequency domain**.

ULF (ultra low frequency)	ms^2^	24 h recordings ≤0.003 Hz
VLF (very low frequency)	ms^2^	24 h and 5-min recordings −0.003–0.04 Hz
LF (low frequency)	ms^2^	24 h and 5-min recordings −0.04–0.15 Hz
HF (high frequency)	ms^2^	24 h and 5-min recordings −0.15–0.4 Hz

## Origin of heart rate variability components

### High frequency oscillations

The cyclic variations in intrathoracic pressure perturbate venous return, cardiac out-put and thus, blood pressure. These changes are sensed by baroreceptors and result in changes in autonomic activity to the heart. These perturbations are mediated via the vagus nerve as atropine administration abolishes high frequency oscillations in heart rate (Akselroad et al., 1981). It seems that a major cause of respiratory sinus arrhythmia (RSA) is a central coupling of respiratory drive to cardiac vagal motor neurons. However, the changes in vagal activity are partly induced by baroreceptor sensing of respiratory oscillations in blood pressure and reflect all components of the baroreflex loop (DeBoer et al., 1987). In addition, factors such as reduced respiratory capacity and body position may alter the amplitude of high frequency oscillations in blood pressure and subsequently the HF component of heart rate signals (Malpas, 2002). Thus, heart rate variability analysis cannot be used for comparisons between different patient groups as there is a need for controlling ventilation for both rate and depth. Moreover, and since there is marked inter-individual variation in the relationship between HRV and parasympathetic effect, differences in HRV between individuals may reflect differences in this relationship, as was postulated by Goldberger (Goldberger et al., 2001). In this respect, this relationship in humans was described by a quadratic function in which there is an initial ascending limb, where HRV increases in parallel with vagal effect until it reaches a plateau level. Beyond this level, HRV decreases with further augmentation of vagal tone, probably due to a saturated HRV response upon intense autonomic stimulation (Goldberger et al., 2001). Finally, age and sex-related differences have also been associated with this variability (Goldberger et al., 2001).

### Low frequency oscillations

The LF component of HRV is probably the most contentious aspect with respect to cardiovascular variability. There are two opposing theories in the literature proposing different potential origins: (1) the central oscillator theory (Montano et al., 1996) and (2) the baroreflex feedback loop theory (Lanfranchi and Somers, 2002). According to the first theory, it is believed that LF oscillations reflect sympathetic tone and are generated by the brain stem circuits. In cats, Montano (Montano et al., 1996) analyzed the dis-charges of single sympathetic neurons located in the rostral ventrolateral medulla and caudal ventrolateral medulla. He observed activity at 0.12 Hz, which was positively correlated with heart rate and blood pressure variability. As the above oscillations remained after sino-aortic and vagal resection, it was assumed that the central nervous system is able to generate such oscillations.

According to the baroreflex feedback loop theory, a change in blood pressure is sensed by arterial baroreceptors, resulting in heart rate adjustment through the central nervous system and via both the fast vagal and the slower sympathetic actions (Lanfranchi and Somers, 2002). At the same time, baroreceptors induce a slow sympathetic withdrawal from the vessels. The delay in the sympathetic branch of the baroreflex in turn determines a new oscillation, which is sensed by the baroreflex and induces a new oscillation in heart rate. It has been also proposed that the LF oscillation arises from the interaction of slow sympathetic and fast vagal responses, where baroreflex buffering of the slow respiratory induced blood pressure oscillations results in resonant low frequency oscillations, due to the delay in the slow conducting sympathetic loop of the baroreflex (DeBoer et al., 1987).

In conclusion, it must be stressed that the low frequency oscillations of heart rate reflect the ability of the individual components of the baroreflex feedback loop to respond to different inputs that can alter the power of such oscillations and they are not just a measure of sympathetic nerve activity.

### Intracardiac origin of HRV

The reasons for reduced HRV during cardiovascular diseases have been debated and two theories have been developed. The first theory focuses on reduction of vagal tone and has been introduced by Akselroad et al. (1981). The second theory developed by Goldberger and colleagues (2002) states that normal physiology has fractal-like properties with high levels of complexity that explain phenomena such as HRV. Its reduction during severe disease reflects a “de-complexification,” mostly attributed to uncoupling between different restorative mechanisms (Godin and Buchman, 1996). In addition, accumulating evidence from both *in vitro* and *ex vivo* experiments support a potential third mechanism (Griffin et al., 2005), which is associated with an intracardiac origin of HRV. According to this hypothesis, sinus atrial node (SAN) cells can be viewed as an amplifier of various input signals (Zaza and Lombardi, 2001). During cardiovascular diseases, an unfavorable metabolic milieu could affect ion channel gating properties or membrane receptor densities, with significant impact upon level and variability of pacemaker activity. In addition, a possible reduced responsiveness of SAN cells to external stimuli could also negatively affect HRV (Zaza and Lombardi, 2001).

Moreover, different clinical studies in heart transplant recipients have found evidence for heart rate fluctuations originating from the heart itself (Hrushesky et al., 1984; Bernardi et al., 1990). Bernardi studied intrinsic mechanism regulating HRV in both transplanted and intact heart during exercise (Bernardi et al., 1990). He found that at peak exercise a non-autonomic mechanism, probably intrinsic to the heart muscle, may determine heart rate fluctuations in synchrony with ventilation, in transplanted as well as in intact hearts. Hrushesky and colleagues (1984) quantified respiratory sinus arrhythmia and found that individuals with a transplanted heart had resting RSA values similar to healthy subjects.

In conclusion, there is marked inter-individual variation between HRV response and different levels of autonomic stimulation. Basal autonomic activity, age and sex differences, alterations in expression of ion channel activity or autonomic receptors could be responsible for individualized curves, relating autonomic effects to HRV (Eckberg, 1997; Goldberger et al., 2001). In addition, LF/HF ratio has been criticized as an indirect measure of sympathovagal balance, reflecting rather autonomic fluctuations and not absolute measures of autonomic nerve traffic (Eckberg, 1997; Billman, 2013). Thus, interpretation of different studies investigating HRV alterations in different groups of patients should be cautious since variability in time of recordings and methods for HRV analysis, as well as heterogeneity of studying population, limit generalization of their findings.

## Clinical implications of altered heart rate variability

The first large prospective population study that reported the significant prognostic value of low HRV after an acute myocardial infarction was the Autonomic Tone and Reflexes After Myocardial Infarction Study (ATRAMI) (La Rovera et al., 1998), and included 1284 patients with a recent (<28 days) myocardial infarction. A 24 h Holter recording was done to quantify HRV (using SDNN values) and ventricular arrhythmias. Low values of HRV (SDNN < 70 ms) carried a significant multivariate risk of cardiac mortality. Furthermore, the association of low SDNN with left ventricular ejection fraction (LVEF) <35% carried a relative risk of 6.7, compared with patients with LVEF above 35%. Investigators from the Framingham Heart Study (Tsuji et al., 1994) computed HRV time and frequency domain measures in 736 patients and correlated them with all-cause mortality during 4 years of follow-up. They concluded that HRV offers prognostic information independent of that provided by traditional risk factors.

During the Zutphen study (Dekker et al., 1997), 885 middle-aged (40–60 years old) and elderly Dutch men (aged 65–85) were followed from 1960 until 1990, whereas SDNN was determined from the resting 12-lead ECG. It was shown that low HRV is predictive of mortality from all causes, indicating that it can be used as an index of compromised health in the general population. It seems that the predictive value of low HRV is independent of other factors, such as depressed left ventricular ejection fraction and presence of late potentials (Kleiger et al., 1987; Lombardi et al., 1987). In addition, retrospective ECG data analysis from 127 patients included in the Veterans Affairs' Survival Trial of Antiarrhythmic Therapy in Congestive Heart Failure (CHF) (Bilchick et al., 2002) demonstrated that CHF patients with SDNN <65.3 ms had a significantly increased risk of sudden death. Moreover, this study demonstrated that every 10 ms increase in SDNN conferred a 20% decrease in risk of mortality.

## Heart rate variability and inflammatory biomarkers in cardiovascular diseases

The relationship between HRV and inflammation has been studied mainly in patients with acute or stable CAD, CHF and metabolic syndrome with impaired glucose tolerance (Brunner et al., 2002). The inflammatory biomarkers that were used included C-reactive protein (CRP), TNF-α, IL-6 and white blood cell count (WBC).

### Patients with coronary artery disease

Hamaad et al. (2005) tested the association between time and frequency domain indices of HRV and circulating IL-6, high sensitivity CRP (hs-CRP) and white cell counts, in a sample of 100 patients with proven acute coronary syndrome. In addition, they compared these metrics with healthy controls (*n* = 49) and estimated possible relationships on repeated measures at 4 months in recovery (*n* = 51). They found modest negative correlations between all inflammatory biomarkers and mainly SDNN, VLF and LF power. The strongest associations were seen between WBC and SDNN (*r* = −0.351). However, relationships did not persist on multivariate analyses after a 4-month period. According to the authors, the correlations were observed largely among HRV indices reflecting sympathetic activity, suggesting that the inflammatory response in acute coronary events may be associated with sympathetic activation instead of vagal withdrawal. Furthermore, leukocytosis observed in these patients seems to be a potential source of pro-inflammatory cytokines within the atheromatous plaque and might induce a potential rupture.

In another study, Lanza and colleagues (2006) assessed HRV and measured CRP serum levels within 24 h of admission in 531 patients with unstable angina pectoris. They found a significant negative correlation between CRP levels and all HRV metrics derived from both time and frequency domain, with the highest correlation coefficient with SDNN and VLF. After categorizing patients into 4 subgroups according to CRP quartile levels, significantly lower HRV values were found in the upper CRP quartile. The subsequent multivariate analysis revealed that SDNN and VLF were the most significant predictors of increasing CRP, whereas CRP was a strong predictor of impaired ANS activity as well.

In a study including patients with suspected CAD, Madsen et al. (2007) enrolled 269 subjects referred for elective coronary angiography. They found that SDNN of heart rate signals was significantly higher in the lower CRP quartile compared to the upper one, whereas associations were stronger for patients with a previous myocardial infarction and with significant coronary stenoses.

In a similar study (Nolan et al., 2007), a negative correlation between CRP and HRV frequency components was reported, whereas a decreased HF power (reflecting vagal tone) in the high CRP quartile, compared to the lowest one, was found. In the study by Janszky and colleagues (2004) that included only female patients who survived hospitalization for acute myocardial infarction and were evaluated 1 year after the event, levels of IL-6 showed an inverse relation with all HRV frequency measures, except for HF. However, this relationship, as well as the association between CRP levels and IL-1 receptor antagonist (IL-1ra) with HRV indices was non-significant. Psychari et al. (2007) also reported a strong inverse association between CRP and several HRV indices (SDNN, HF, and LF) in post-MI patients and after adjustment for left ventricular function.

Recently, von Känel et al. (2011) investigated the association between HRV measured in the time domain, CRP, IL-6 and fibrinogen, in a cohort of 862 subjects recruited from the Heart and Soul Study, which assessed health outcomes in 1.024 outpatients with stable CAD. They found that SDNN was inversely and significantly associated with inflammatory indices, after adjustment of all covariates.

### Patients with heart failure

In 2001, Aronson et al. (2001) evaluated for the first time the relationship between HRV metrics derived from both time and frequency domains and different biomarkers, such as IL-6, TNF-α, and serum levels of norepinephrine, in 64 patients admitted for decompensated chronic heart failure. TNF-α levels did not correlate with any of the HRV indices. However, IL-6 was inversely correlated with SDNN (*r* = −0.36), with total power of heart rate signals and ULF (*r* = −0.37 and *r* = −0.43, respectively). No correlation was found between IL-6 and time (pNN50 and RMSSD) or frequency domain (HF power) indices of vagal activity.

Malave et al. (2003) examined HRV in relation to circulating levels of TNF-α, TNF-α receptors and norepinephrine in 10 controls, 15 patients with mild CHF and 14 subjects with moderate heart failure. There was a significant inverse linear correlation between increased levels of all biomarkers and SDNN, LF and HF power among CHF patients. In addition, LF power was more closely correlated with circulating levels of TNF-α than was the HF component, whereas multiple linear regression analysis showed that TNF-α was a stronger predictor of reduced HRV than was the circulating levels of norepinephrine. The authors concluded that over-expression of TNF-α and subsequent loss of β-adrenergic responsiveness contributes to the decrease in HRV, observed in heart failure. According to findings from experimental studies (Chung et al., 1990), TNF-α might inhibit β-adrenergic signal transduction through either activation of Gi proteins or impairment of activation of Gs proteins, something that could be viewed as an adaptive mechanism in the early stages of CHF, protecting cardiac myocytes from the deleterious actions of catecholamines. However, in the more advanced stages of the disease, this mechanism could become maladaptive, leading to a reduction in cardiac output (Mann et al., 1992).

Finally, in a small prospective study that included 34 patients with CHF followed for a 2-year period with monthly CRP measurements and 24-h Holter recordings, it was shown that five unexpected deaths that occurred were preceded by progressive increases in both CRP serum levels and autonomic dysfunction (low HRV indices) (Shehab et al., 2004).

### Healthy controls

As a part of the Copenhagen Holter study, that assessed the value of 24-h Holter recording in the risk assessment of men and women aged 55, 60, 65, 70, and 75 years with no apparent heart disease, Sajadieh et al. (2004) investigated the associations between time domain components of HRV, CRP and WBC in 643 healthy men and women. They found that SDNN was negatively correlated with smoking, inflammatory indices, blood sugar, triglyceride concentration, female gender and diabetes. Moreover, in multivariate regression analysis, increased heart rate and reduced HRV were significantly related to white blood cell count and CRP. The reduction of SDNN was attributed to sympathetic predominance, whereas lack of any association between inflammation and pNN50, which is considered as a marker of vagal activity, indicates that reduced HRV is mainly due to increased sympathetic activity rather than vagal withdrawn.

From the Whitehall II cohort, a multicenter epidemiologic investigation of over 5000 subjects, two studies (Owen and Steptoe, 2003; Sloan et al., 2007) used sub-samples to examine the relation between HRV indices derived from the frequency domain and inflammation, in healthy subjects. In the first study, Sloan found in a sample of 757 people, an inverse correlation between CRP and IL-6 with both LH and HF components of HRV power spectrum. In the second study, Owen and Steptoe did not find any association between IL-6, TNF-α and time domain measures of HRV, in a group of 211 healthy adults.

Other investigators (Albert et al., 2002) reported a strong positive association between CRP levels and the long-term risk of sudden cardiac death, in case-control analysis among healthy individuals, followed for 17 years in the Physician's Health Study. Men in the upper CRP quartile had a 2.8 fold increased risk of sudden cardiac death compared to men in the lower quartile. According to the authors, a low-grade inflammation involved in atherosclerosis shifts ANS balance toward sympathetic activation, making individuals more prone to ventricular arrhythmias and sudden cardiac death.

The Twins Heart Study (THS) (Goldberg et al., 2002) was an investigation of psychological, biological and behavioral risk factors for subclinical cardiovascular diseases in 7.369 middle-aged male-male twin pairs, who served in the United States military during the Vietnam War. From this registry, a cohort of 264 twins free of symptomatic CAD was examined by Lampert et al. (2008), for assessing possible associations between HRV, CRP, and IL-6. They found an inverse relationship between frequency domain HRV metrics (except for HF) and both CRP and IL-6. These associations persisted after adjustment for other traditional CAD risk factors, such as smoking, hypertension, diabetes, high-density lipoprotein (HDL) and depression.

A significant confounding factor of HRV analysis that has to be considered in these studies includes the presence of depressive symptoms and anxiety. It has been estimated that ~12–20% of hospitalized cardiac patients suffer from major depression, whereas 15% of subjects following acute myocardial infarction exhibit a posttraumatic stress disorder (Frasure and Lesperance, 2006; Garder and von Karel, 2006; Pizzi et al., 2008). In a 2-year follow up prospective observational study, Pizzi investigated the relation between time domain HRV indices, IL-6, TNF-α, CRP and depression in a cohort of 415 subjects free of CADs, with at least two CAD risk factors (age, male gender, current smoking, hypertension, dislipidaemia). All HRV and inflammatory indices were significantly associated with depression. Logistic regression further showed that depressive individuals were more likely to have a higher CRP and IL-6 and altered HRV (lower SDNN).

In a recent study of Kop and colleagues (2010) who recruited 908 patients, free of CAD, for a median follow-up period of 13.3 years, it was demonstrated that among depressed participants, HF power of HRV was negatively correlated with CRP (*r* = −0.205), IL-6 (*r* = −0.233) and WBC (*r* = −0.292). Moreover, depression was associated with high IL-6 serum levels and increased cardiovascular mortality risk. In conclusion, in patients without heart disease depression seems to be associated with HRV imbalance and inflammation.

Table 3 summarizes the majority of clinical studies that have evaluated the relationship between different inflammatory biomarkers and HRV metrics, in patients with different cardiovascular diseases.

**Table 3:** **Summary of several clinical studies investigating a possible association between HRV indices and inflammation in patients with CAD, CHF and healthy individuals**.

**References**	**Study population**	**Duration and HRV measures**	**Inflammatory indices**	**Results**
Hamaad et al., 2005	100 patients with acute CAD vs. 29 healthy controls	20 min time, time and frequency domain	CRP, IL-6	Negative correlation with SDNN, VLF and LF
Lanza et al., 2006	531 patients with unstable CAD	24-h time, time and frequency domain	CRP	Inverse correlation between CRP with SDNN and VLF
Madsen et al., 2007	269 patients with suspected CAD	24-h time, time domain	CRP	Upper CRP quartile negatively correlated with SDNN
Nolan et al., 2007	29 patients with CAD	5-min time, frequency domain	CRP	HF power decreased in high CRP group
Psychari et al., 2007	98 patients with acute CAD (post-MI)	24-h time, time and frequency domain	CRP	Inverse relation between CRP and SDNN, HF and LF power
von Känel et al., 2011	862 patients with CAD	24-h time, time domain	CRP, IL-6, fibrinogen	Inverse association between CRP, IL-6 and SDNN
Aronson et al., 2001	64 patients with CHF	24-h time, frequency domain	TNF-α, IL-6	IL-6 inversely correlated with SDNN and ULF power
Malave et al., 2003	10 healthy controls, 15 patients with mild CHF, 14 patients with moderate CHF	24-h time, frequency domain	TNF-α, TNF soluble type 1 and 2 receptors	Inverse correlation between inflammatory measures, SDNN, LF and HF
Sajadieh et al., 2004	643 subjects without CHF	24-h time, time domain	CRP, WBC	Inverse correlation between SDNN with CRP and WBC SDNN predictor of CRP
Sloan et al., 2007	757 young healthy adults	10-min time, frequency domain	CRP, IL-6	CRP and IL-6 inversely correlated with HF and LF
Owen and Steptoe, 2003	211 healthy subjects	20–30 min time, time domain	TNF-α, IL-6	No relation between both TNF-α and IL-6 with HRV
Lampert et al., 2008	264 healthy twins individuals	24-h time, frequency domain	CRP, IL-6	Inverse relation between CRP and IL-6 with all HRV frequency metrics (except for HF)

Abbreviations *CRP* *C-reactive protein* *CAD* *coronary artery disease* *CHF* *congestive heart failure* *MI* *myocardial infarction* *WBC* *white blood cell count.*

## Heart rate variability and systemic inflammation in critical illness

The presence of sympathetic overactivity, autonomic dysfunction, inappropriately increased heart rate, insulin resistance and in some cases, cardiomyopathy with reduced cardiac contractility, has also been observed during severe sepsis and multiple organ failure (Muller-Werdan et al., 2006). However, in cardiac patients sympathetic activity dominates over vagal tone where in septic patients both branches of ANS are attenuated (Muller-Werdan et al., 2006). For these reasons, it has been hypothesized that during critical illness except for ANS impairment, a defective signal transduction at the level of pacemaker cells could also account for observed differences between cardiac and septic patients (Fairchild et al., 2009).

Alterations in HRV during septic shock and multiple organ dysfunction syndrome (MODS), have been reported from different research groups (Goldstein and Buchman, 1998; Goldstein et al., 1998; Seely and Christou, 2000). In this respect, Goldstein et al. (1998) found that both increased total variability and LF power were associated with recovery and survival, whereas a decrease in total power, LF/HF and LF power correlated with severity of illness and mortality in septic patients, 48 h after being admitted to the Intensive Care Unit. In an animal study of experimental endotoxemia, induced by administration of lipopolysacchraride (LPS, endotoxin derived from the cell wall of Gram-negative bacteria) Fairchild and colleagues demonstrated a strong inverse correlation between SDNN and total power of RR time series and peak concentrations of different cytokines, 3–9 h post-LPS (Fairchild et al., 2009). The same results were found after administration of recombinant TNF-α. It was suggested that mechanisms responsible for decrease in HRV could be related with effects of LPS and/or cytokines on various ion channels.

Tateishi et al. (2007) investigated the relationships between HRV and interleukin 6 upon admission in a cohort of 45 septic patients and they found that IL-6 exhibited significant negative correlations with both LF and HF power values. These findings indicate a possible association between low HRV indices and hyper-cytokinemia.

**Figure 1:**
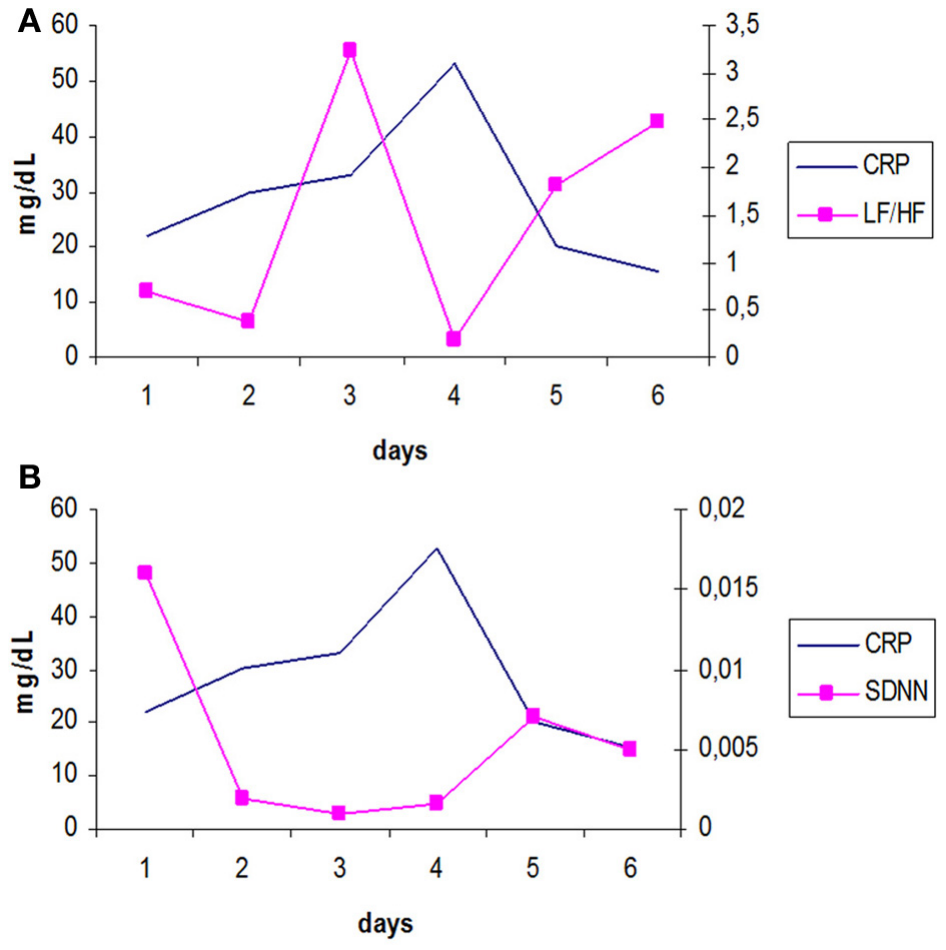
**(A)** Longitudinal trends over time of mean values of CRP and LF/HF ratio, reflecting sympathovagal balance, for patients with SOFA > 10, during the 6 days of study period. [log transformed data, adapted from Papaioannou et al. (2009)]. It appears that LF/HF changes inversely with CRP. **(B)** Longitudinal trends over time of mean values of CRP and SDNN (secs), for patients with SOFA > 10, during the 6 days of study period. [log transformed data, adapted from Papaioannou et al. (2009)]. There is a progressive increase in SOFA score from day 1 until day 4 (development of septic shock) and a subsequent downward shift in its values. At the same time, the variability of heart rate signals estimated with SDNN seems to be significantly reduced during the development of septic shock.

In another study Papaioannou et al. (2009), we investigated possible associations between different HRV indices and various biomarkers of inflammation, in 45 septic patients and during the first 6 days of their stay in the ICU. We daily assessed HRV in time (SDNN) and frequency domain (LF, HF and LF/HF) and measured C-reactive protein, Interleukin 6 and 10 serum levels in two groups of patients. The first group included subjects suffering from sepsis with mean Sequential Organ Failure Assessment score of severity of illness (SOFA) ≤ 10 (*n* = 25) and the second group included patients with septic shock (SOFA > 10, *n* = 20). This study found in the group of patients with SOFA > 10, statistically significant inverse correlations between CRP and LF/HF ratio (*r* = −0.61), (Figure 1A) and positive correlations with HF (*r* = 0.80). At the same time, IL-10 proved to be significantly correlated with HF and SOFA score in a positive way and with LF, LF/HF and SDNN in a negative way. Finally, the total variability of heart rate signals (SDNN) was found to be negatively correlated with both CRP (*r* = −0.79) and SOFA score (*r* = −0.84) (Figure 1B). IL-6 was not significantly correlated with any HRV parameter. It is possible that, the pleuripotency of this cytokine could be responsible for our results since IL-6 can behave as both a pro-inflammatory activator (induces the production of CRP) and inhibitor (limits Tumor necrosis factor α and IL-1β secretion), at the same time (Chrousos, 1995).

These findings suggest that reduction in HRV and LF/HF is related with an augmented pro- and anti-inflammatory response during sepsis, especially in more severely ill patients. Furthermore, severity of illness is positively associated with HF and IL-10 serum concentrations and changes inversely with variability of heart rate signals. In this respect, elevated levels of IL-10 have also been found in trauma patients who developed sepsis and multiorgan failure (Sherry et al., 1996).

In conclusion, it seems that critical illness and high cytokine levels are associated with reduced HRV, however, existing literature does not elucidate whether loss of HRV is related to an endotoxin effect at the level of ANS output, baroreflex sensitivity or the pacemaker cell itself. However, results from a prospective study in a group of 40 healthy adults who received a single intravenous bolus of 2 ng/kg LPS, suggested that there is no relationship between basal cardiac ANS activity, and the inflammatory response (Kox et al., 2011). Thus, no association was found between frequency components of HRV that were determined hourly and until 8 h after LPS administration and different pro- and anti-inflammatory cytokines, measured at various time points. According to the authors, vagus nerve innervation of the heart does not reflect outflow to other organs, such as the spleen, one of the major cytokine-producing organs (Tracey, 2002, 2007). As basal vagal input to the spleen may be different from vagal input to the heart, HRV might not be an appropriate method to assess activation of the cholinergic anti-inflammatory pathway (Kox et al., 2011). These findings strengthen the notion that autonomic outflow cannot be regarded as a general response, but appears to be organ-specific. In this respect, results from different studies discussed so far lack generalization and robustness due to different study populations and design, interspecies differences and potential impact of severity of disease, sedation or mechanical ventilation upon HRV (Goldstein and Buchman, 1998). However, it appears that an inverse association between inflammation and total variability of heart rate signals could be found in the most severe cases.

## Potential therapeutic implications

Different clinical trials have shown that fatty acids from fish oil can be considered as powerful disease-modifying nutrients in patients with acute lung injury, sepsis and cardiovascular diseases (Christensen et al., 1999; Abuissa et al., 2005; Pontes-Arruda et al., 2006; Singer et al., 2006). Particularly, feeding with the very-long chain, ω-3 polyunsaturated fatty acids (PUFAs) eicosapentaenoic acid (EPA) and docasahexaenoic acid (DHA) has been found to inhibit the activity of the pro-inflammatory transcription factor nuclear factor kB (NF-kB) and subsequently, to attenuate the production of different cytokines, chemokines and other effectors of innate immune response (Singer et al., 2008). In the cardiovascular literature, it has been shown that oral supplementation of ω-3 PUFAs increase instantaneous HRV, reduce LF/HF ratio and confer protection against ischemia-induced ventricular tachycardia and sudden cardiac death (Abuissa et al., 2005). Moreover, Christensen (Christensen et al., 1999) demonstrated that fish oil feeding can induce an incorporation of DHA into the membranes of granulocytes, which is associated with a dose-response increase in HRV (SDNN), something that may protect against serious ventricular arrhythmias. Such effects of fish oil reflect an enhanced efferent vagal activity via a central-acting mechanism, due to a possible suppression of pro-inflammatory cytokines that have been found to inhibit central vagal neurons (Singer et al., 2008).

Never-the-less, different interventional studies on ω-3 PUFAs and HRV in patients with heart disease have found inconsistent results, with only 8 out of the 20 trials published so far, supporting a beneficial effect on HRV (Christensen, 2011). Indeed, Mozaffarian et al. (2008) reported that individuals with the highest fish consumption (≥5 meals/week) only exhibited 1.5 ms greater HRV compared to those with the lowest fish consumption and further that this modest reduction in HRV was associated with only a 1.1% reduction in the relative risk for sudden cardiac death. Reasons for such inconsistency might include heterogeneous populations, limited sample sizes or different study protocols with variable administered doses of ω-3 PUFA and length of intervention. Furthermore, different methods of measurement of HRV with variable time of recordings could be an additional confounder. Finally, an animal study with administration of ω-3 PUFAs in rabbits showed that a reduction in pacemaker funny current rather than an alteration in autonomic neural regulation was responsible for heart rate reduction and increase in HRV (Verkerk et al., 2009). However, such experiments were performed in denervated hearts, excluding a potential impact of autonomic tone on HRV. More recently, the HRV response to physiological challenges was not altered by dietary ω-3 fatty acids in conscious intact preparations; data that further suggest that these lipids elicited alterations in pacemaker rate rather than cardiac autonomic regulation (Billman and Harris, 2011; Billman, 2012).

Recent evidence suggests that HMG-CoA reductase inhibitors (statins) have pleiotrophic mechanisms in patients with heart failure, such as ANS output modulation (Lefer, 2002). Experiments with animal models of heart failure have found decreased sympathetic activation and autonomic balance restoration with statins, using HRV analysis (Pliquett et al., 2002). In a cross-over study of HRV in 30 patients with hyperlipidemia (Welzig et al., 2003), pravastatin administration induced a significant increase in HF power of ECG signals, whereas others (Vrtovec et al., 2005) found that administration of 10 mg of atorvastatin for 3 months, was associated with significant increase in SDNN and RMSSD, in 80 patients with CHF and hyperlipidemia. Moreover, cholesterol lowering was not correlated with HRV changes, suggesting another mechanism than that of lipid-lowering of statins. In this context, Gao et al. (2005) showed that in experimental heart failure states there is intense free radical production and up-regulation of angiotensin receptors, in autonomic areas of the brain. Moreover, simvastatin therapy was proven to inhibit angiotensin II and superoxide pathways in the RVLM of pacing-induced heart failure rabbits, leading to an abolished renal sympathetic nerve activity (Gao et al., 2005).

Different experimental studies have shown that catecholamines, except from increasing cardiac contractility and heart rate via interaction with beta adreno-receptors, may induce myocardial damage by calcium overload and subsequent cell necrosis, upon excessive β-adrenoreceptor stimulation (Opie et al., 1985; Mann et al., 1992). Although, toxic cardiac effects of catecholamines have been recognized since 1907, Rona et al. (1959) was the first who observed that isoproterenol injection into rats produced “infarct-like” myocardial necrosis, in the absence of coronary artery lesions. He proposed the theory of “relative hypoxia” as a pathophysiological mechanism, suggesting a possible imbalance between oxygen demand and blood flow, after excessive adrenergic stimulation. Fleckenstein (1971) thought that calcium overload was the result of catecholamine-mediated cell injury, due to extensive activation of Ca-dependent ATPases and subsequent high energy phosphate deficiency, leading to mitochondrial impairment. On the contrary, Opie and co-workers (1985) were the first who demonstrated that catecholamine cell injury was due to calcium overload, mediated by the β-adrenoreceptor. More recent studies have confirmed previous results and have also demonstrated that other mechanisms could be responsible as well, for catecholamine-induced cell injury, such as increased fibrosis of the left ventricle with associated hypertrophy (Briest et al., 2001) or myocardial cellular apoptosis (Commural et al., 1998).

Since heart failure is associated with a sympathetic up-regulation and parasympathetic withdrawal, β blockers have been used to modify the effects of augmented sympathetic tone and restore autonomic imbalance. In this respect, Goldsmith et al. (1997) showed that administration of carvedilol for 4 months was associated with a significant increase in HF power, in patients with CHF under digoxin and angiotensin-converting enzyme (ACE) inhibitors. Similar results were found in post-MI subjects by Lampert et al. (2003), after treatment with propranolol for 6 weeks. An elevation in HF, which reflects restoration of sympatho-vagal balance, was found to increase final outcome (LVEF, exercise capacity, death and CHF development) in both studies.

Recent evidence suggests that a primary site of attenuated vagal control on the heart occurs at the level of the parasympathetic gaglion (Bibevski and Dunlap, 2011). Thus, cervical vagus nerve stimulation (VNS) has been recently assessed as an “add-on” therapy to optimal medical management of CHF. Li and colleagues (2004) were the first who found that VNS performed for 10 s every minute, in rats developed HF after anterior myocardial infarction, improved significantly left ventricular function and decreased mortality from 50 to 14%, in comparison with sham treated animals. Zhang et al. (2009) recently demonstrated in a canine model with high rate ventricular pacing induced HF, that VNS over 12 weeks was able to reduce left ventricular end systolic and end-diastolic volumes and increase LVEF significantly. In addition, HRV was significantly improved in VNS dogs whereas plasma norepinephrine and CRP levels were markedly attenuated with VNS treatment. Finally, vagal stimulation has also been found to limit infarct size and inflammatory response to myocardial ischemia and reperfusion in male rats that underwent myocardial ischemia for 30 min and reperfusion for 24 h (Calvillo et al., 2011). According to the authors, the anti-inflammatory and anti-apoptotic properties of the nicotinic pathway were the primary underlying mechanism in the VNS-treated animals.

Based on the results of VNS in animal models of HF, Schwartz et al. (2008) and De Ferrari et al. (2011) were the first who assessed feasibility and safety and tested possible efficacy of chronic VNS in HF patients with New York Heart Association (NYHA) class II-IV symptoms. In a two-staged study, (8-patients feasibility phase plus 24-patients safety and tolerability phase) they used CardioFit, a right cervical VNS implantable system delivering pulses synchronous with heart beats through a multiple contact bipolar cuff electrode. VNS was started 2–4 weeks after implant and patients were followed 1, 3, and 6 months thereafter. VNS was well tolerated whereas, there was a significant improvement in NYHA class and left ventricular end-systolic volume. Moreover, a significant increase in HRV, estimated with pNN50 and slightly but significantly reduced heart rate were found 6 months after VNS onset (Schwartz et al., 2008; De Ferrari et al., 2011).

As a consequence of such preliminary results, a pivotal multicenter international clinical trial, the INOVATE-HF study, has been recently designed to assess safety and efficacy of VNS in 650 CHF patients from 80 sites, with NYHA class III symptoms, sinus rhythm and QRS width less than 120 ms, using the CardioFit system (Hauptman et al., 2012). Thus, in case of significant decrease in mortality, vagal stimulation will add significant value to current medical therapy in a narrow spectrum of patients with heart failure, through restoration of sympatho-vagal balance. Never-the-less, a lot of questions remain to be addressed, such as optimal stimulation mode (i.e., right vs. left vagus nerve stimulation, continuous vs. pulse-synchronous stimulation etc) or effective and tolerable VNS dose, before adopting this new non-pharmacologic treatment to our therapeutic armamentarium.

## Conclusions and future suggestions

Different experimental studies have established an inter-relation between ANS output and inflammatory regulation, whereas the discovery of the “cholinergic anti-inflammatory pathway” has expanded our understanding of how the nervous system modulates the inflammatory response through an immunoreflex. Furthermore, clinical data from large epidemiological studies involving patients with CAD, heart failure and healthy subjects with increased risk factors for heart diseases suggest that there is a rather weak or moderate association between inflammation and ANS activity, estimated through HRV analysis. Finally, investigation of HRV alterations during critical illness, such as sepsis and MODS, has demonstrated loss of variability of heart rate signals that is inversely correlated with immune response, particularly in most severe cases.

Early and more accurate monitoring of cardiovascular patients, particularly in the early stages of life-threatening illnesses through continuous automated detection of abnormal variability of heart rate signals, could alert clinicians to impending clinical deterioration and allow earlier intervention. In this respect, the combination of structural indices, such as the left ventricular ejection fraction, with autonomic function indices derived from heart rate variability analysis has been proposed as the state-of-the-art method for risk assessment among patients with acute myocardial infarction or severe congestive heart failure (Priori et al., 2001). However, in-consistent findings from different studies assessing the relationship between HRV frequency components and inflammation limit adoption of HRV analysis as an indirect estimator of inflammatory response, since there is a marked heterogeneity in study protocols, time and methods of HRV measurement and patients' characteristics. Moreover, HRV analysis does not simply reflect sympathetic/parasympathetic balance, since HRV data can be influenced by artifacts related to differences in breathing characteristics, genetic factors or basal autonomic tone. Thus, any change in LF/HF ratio may correspond to central, baroreflex or cellular membrane effects of different stimuli during severe stress. Furthermore, sympathetic outflow can either induce or inhibit inflammatory activity, whereas HF component might fail to reflect vagal inputs upon different organs, such as the spleen, which are major cytokine producers. In addition, the explanatory power of HRV analysis is affected by circadian variability as well as by the estimation of inflammatory activity through biomarkers' measurements from a single blood sample (Haensel et al., 2008). Finally, lack of signals' stationarity (stable statistical properties) during measurements and the strong association between HRV data and inflammation that has been demonstrated among the most severely ill patients could reflect a non-linear relationship between ANS and inflammatory response. Thus, it has been suggested that newer methods derived from chaos theory should be implemented to assess ANS output, such as fractal and detrended fluctuation analysis (DFA) of heart rate signals (Goldberger, 1996). In addition, standardization of experimental protocols and methods used for the estimation of HRV is urgently needed, in order to allow comparisons among different studies.

In conclusion, the enormous complexity of neuro-immunological interactions cannot be captured by simple measurements, such as HRV analysis. A rather multi-parameter monitoring of ANS, through different estimators of heart rate variability and complexity has been suggested for assessment of ANS output (Goldberger, 1996). In any case and since HRV metrics are not enough for differentiating between patho-physiological states (poor specificity) or between patients (poor sensitivity), longitu-dinal alterations over time of both HRV and inflammatory markers on an intra-individual basis must be tested for establishing a potential added value of HRV analysis, as an indirect estimator of inflammatory response (Papaioannou et al., 2009).

### Conflict of interest statement

The authors declare that the research was conducted in the absence of any commercial or financial relationships that could be construed as a potential conflict of interest.

## Abbreviations

ACE: angiotensin-converting enzyme
ACTH: adrenocorticotropin hormone
ANS: autonomic nervous system
ATRAMI: Autonomic Tone and Reflexes After Myo-cardial Infarction Study
CAD: coronary artery disease
CHF: congestive heart failure
CNS: central nervous system
CRP: C-reactive protein
DHA: docasah-exaenoic acid
DMN: dorsal motor nucleus of the vagus
DVC: dorsal vagal complex
ECG: electrocardiogram
EPA: eicosapentaenoic acid
FFT: Fast Fourier trans-formation
HPA: hypothalamo-pituitary-adrenal axis
HRV: heart rate variability
HF: high frequency
LF: low frequency
LVEF: left ventricular ejection fraction
MODS: multiple organ dysfunction syndrome
NYHA: New York Heart Association
NTS: nucleus tractus solitaries
pNN50: proportion derived from dividing NN50 (number of interval differences of successive intervals greater than 50 ms) by the total NN intervals
PSD: power spectrum density
PUFA: polyunsaturated fatty acids
PVN: paraventricular nucleus
RMSSD: square root of the mean squared differences of successive intervals
RVLM: rostral ventrolateral medulla
TNF-α: tumor necrosis factor α
SAN: sinus atrial node
SDNN: standard deviation of the normal-to-normal intervals which is the square root of the variance
SD: standard deviation
SNS: sympathetic nervous system
THS: Twins Heart Study
ULF: ultra low frequency
VLF: very low frequency
WBC: white blood cell count.
